# Metal–Organic Framework-Capped Gold Nanorod Hybrids for Combinatorial Cancer Therapy

**DOI:** 10.3390/molecules29102384

**Published:** 2024-05-18

**Authors:** Chong Zhao, Hongxiang Liu, Sijun Huang, Yi Guo, Li Xu

**Affiliations:** Key Laboratory for Molecular Enzymology and Engineering, The Ministry of Education, National Engineering Laboratory for AIDS Vaccine, School of Life Sciences, Jilin University, Changchun 130012, China; zhaochong23@mails.jlu.edu.cn (C.Z.); hxliu15@mails.jlu.edu.cn (H.L.); huangsj17@mails.jlu.edu.cn (S.H.)

**Keywords:** combinatorial cancer therapy, MOF, DOX, photothermal, ROS

## Abstract

Recently, nanomaterials have attracted extensive attention in cancer-targeting therapy and as drug delivery vehicles owing to their unique surface and size properties. Multifunctional combinations of nanomaterials have become a research hotspot as researchers aim to provide a full understanding of their nanomaterial characteristics. In this study, metal–organic framework-capped gold nanorod hybrids were synthesized. Our research explored their ability to kill tumor cells by locally increasing the temperature via photothermal conclusion. The specific peroxidase-like activity endows the hybrids with the ability to disrupt the oxidative balance in vitro. Simultaneously, chemotherapeutic drugs are administered and delivered by loading and transportation for effective combinatorial cancer treatment, thereby enhancing the curative effect and reducing the unpredictable toxicity and side effects of large doses of chemotherapeutic drugs. These studies can improve combinatorial cancer therapy and enhance cancer treatment.

## 1. Introduction

Currently, the primary cancer treatment methods include surgery, chemotherapy, and radiotherapy [1]. The inability to completely remove all cancer cells during surgery increases the possibility of cancer recurrence [2]. Chemotherapy and radiotherapy generally have limitations, such as systemic toxicity and side effects. In response to these challenges, researchers have explored novel approaches for cancer therapy, including the development of nanomaterial-based strategies that can offer better personalized treatments [3]. 

Nanomaterials can selectively target and kill cancer cells while sparing healthy tissues. Among these materials, gold nanorods (GNRs) and metal–organic frameworks (MOFs) have emerged as significant candidates because of their unique properties and potential for medical applications [4,5]. GNRs are particularly notable for their photothermal capabilities; they can convert near-infrared light into heat and effectively kill cancer cells through hyperthermia [6]. Synthesized GNRs are nanomaterials with good and stable physical and chemical properties and photothermal effects. They offer good biocompatibility, and their surfaces are easily modified, which can be enriched in tumor cells by cell uptake [7]. Developing multifunctional GNR platforms that integrate imaging, drug delivery, photothermal therapy, and other therapeutic modalities can offer comprehensive cancer treatment strategies [8]. Designing versatile GNR systems with tunable properties for personalized medicine approaches may be a promising application. After combining multiple therapies, the therapeutic effect was greatly improved, as composite materials with the ability to load drug molecules further strengthened their therapeutic effects. 

By contrast, MOFs are crystalline materials composed of metal ions or clusters connected by organic ligands, forming porous structures with high surface areas and tunable properties. MOFs have emerged as a promising class of materials for cancer therapy applications, including drug delivery or imaging [9,10]. The controlled pore size and surface chemistry characteristics of MOFs allow for the encapsulation and release of therapeutic agents, thus offering potential advantages for cancer treatment. Owing to the unique redox chemistry, photochemical property, and catalytic activity of copper-based MOFs (Cu-MOFs), they have been extensively explored in biomedical fields. A synergistic approach to combination cancer therapy allows MOF-based hybrids to improve cancer treatment outcomes [11].

Doxorubicin (DOX) is a widely used chemotherapeutic drug for tumor treatment. It belongs to the anthracycline class of medications and is effective against a variety of cancers, including breast, ovarian, and lung cancers. DOX exerts its anticancer effects through multiple mechanisms, including intercalation into DNA, the inhibition of topoisomerase II, the generation of free radicals, and the induction of apoptosis in cancer cells [12]. Despite its potent therapeutic efficacy, the clinical utility of DOX is often limited by its dose-limiting systemic toxicity. Strategies to mitigate systemic toxicity, such as liposomal formulations and polymer-based nanoparticles, have been developed to reduce side effects [13]. Moreover, the combination of photodynamic therapy with chemotherapy can lead to additive or synergistic antitumor effects [14].

The integration of GNRs and MOFs with drugs represents a favorable approach in nanomedicine to harness the multiple effects of photothermal therapy, catalytic action, and drug release. This hybrid system is a combinatorial strategy that can potentially overcome the limitations of conventional cancer therapies by integrating the multiple effects of hyperthermia or low-dose drug delivery within a single platform [15,16].

In this context, the present study aimed to investigate the potential of metal–organic framework-capped gold nanorod hybrids for combinatorial cancer therapy. We synthesized hybrid materials using MOFs and GNRs; characterized their physicochemical properties; and evaluated their drug-loading capacity, encapsulation rate, and drug release, reasonably adjusted to realize drug delivery, reduce side effects, and increase the malignant tumor treatment effect by combining the chemo-photothermal effect in vitro. By exploring the multiple effects of photothermal therapy, chemotherapy, and catalytic action, we aimed to develop novel strategies for precise cancer treatment with enhanced therapeutic outcomes.

## 2. Results

### 2.1. Metal–Organic Framework-Capped Gold Nanorods Hybrid Characterization

**Figure 1:**
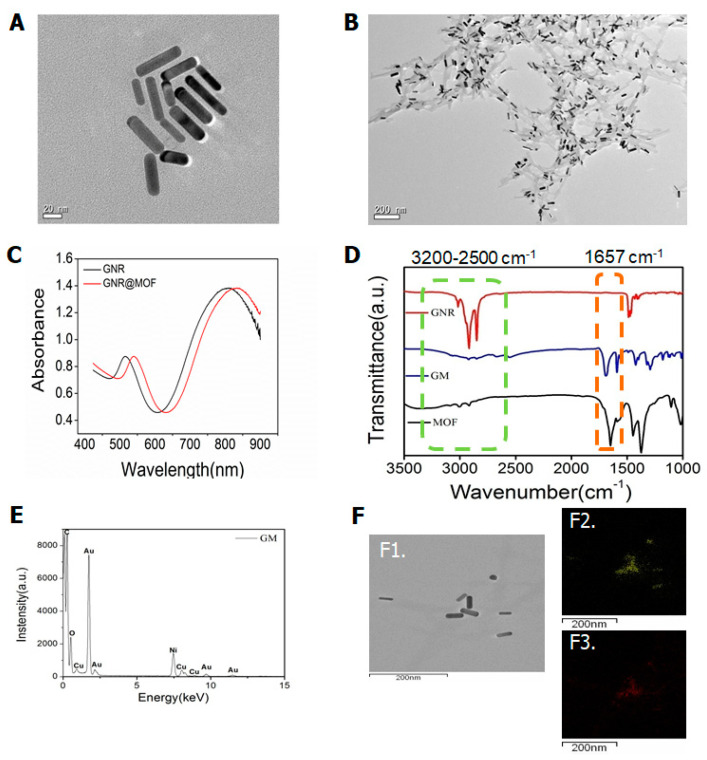
(**A**) Transmission electron microscopy (TEM) image of gold nanorods (GNRs); (**B**) TEM image of gold nanorod coated metal–organic framework (MOF) structures (GM); (**C**) optical extinction spectra of the colloidal Au nanorods stabilized by CTAB and coated with MOF; (**D**) FT-IR spectrum of GNR, GM, and MOF nanomaterials; (**E**) EDS spectrum of GM nanomaterials; (**F**) STEM-EDS elemental mapping of GM, TEM of GM (**F1**), Au (**F2**), Cu (**F3**).

The synthesized gold nanorods existed in a rod-like form with an aspect ratio of 3.7, as characterized by transmission electron microscopy (TEM; Figure 1A). After the formation of the metal–organic framework layer, the core–shell structure of the gold nanorod@MOF (GM) was also confirmed by TEM (Figure 1B). The optical extinction spectrum of the gold nanorods exhibited distinct peaks at approximately 516 and 808 nm (Figure 1C), which originated from the transverse and longitudinal localized surface plasmon resonances (LSPRs) of the gold nanorods, respectively. By contrast, after the deposition of the MOF shell, the transverse LSPR peak underwent a slight red shift, whereas the longitudinal LSPR peak, which was much more sensitive to the environmental refractive index change, showed a distinct red shift from 808 nm to 840 nm (Figure 1C). This remarkable plasmon shift was attributed to the larger refractive index of the MOF compared with that of water [1,17]. Alternatively, gold nanorods are close together in some GM structural regions, which increases the hotspot generation of GNR photothermal conversion [17]. 

To further verify the successful removal of cetyltrimethyl ammonium bromide (CTAB) from the GNR surfaces, infrared spectroscopy (IR) was used to identify the surface composition. We compared the IR spectra of the different nanomaterials. As shown in Figure 1D, the three curves represent the FTIR spectra of the GNR, GM, and MOF nanomaterials. Gold nanorods exhibited strong symmetric and asymmetric C-H stretching at 2900 and 2850 cm^−1^, as well as C-H scissoring at 1430–1470 cm^−1^, which corresponded to the hydrocarbon tail of CTAB [18]. Neither the GM nor the MOF samples exhibited visible IR absorption of these peaks, indicating the absence of CTAB on the surface. 

The most important functional groups employed in MOF preparation here were the stretching vibration peak of C=O at about 1608 cm^−1^ and the C-O stretching at about 1449 and 1323 cm^−1^ belonging to TMA, as shown in the spectra [19]. The absorption peak at the wave number of 1657 cm^−1^ was a redshift phenomenon caused by the coating of Cu-MOF on the gold nanorod surface. Additionally, the 1657 cm^−1^ absorption peak did not exist on the surface of the GNR nanomaterials alone but only appeared after the surface was coated with Cu-MOF (Figure 1D). Overall, these results demonstrate that GM nanomaterials were synthesized. 

The EDS spectra indicated that the GM nanomaterials consisted mainly of Au, Cu, and C and that the peak corresponding to Ni originated from the Ni-supporting grid (Figure 1E). STEM-EDS elemental mapping revealed that Au and Cu were homogeneously distributed and decorated with Cu (Figure 1F).

### 2.2. Drug Loads and Releases 

**Figure 2:**
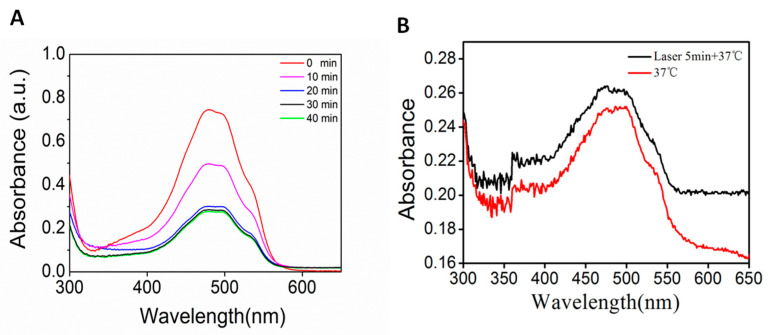
(**A**) UV–Vis absorption spectrum of supernatant drug after incubation with GM from 0 to 40 min; (**B**) UV–Vis absorption spectrum of released drug at 37 °C or with laser irradiation.

To determine the GM nanomaterial’s doxorubicin carrying capacity and encapsulation rate, we used UV–Vis absorption spectroscopy. The doxorubicin standard curve (Figure S1) showed a good linear relationship (*y* = 0.225*x* + 0.001, R2 = 0.998) and was used to evaluate the doxorubicin loading and release capacity. The maximum drug loading was reached after 30 min of incubation (Figure 2A), and according to the carrying amount and drug-loading formula, the encapsulation rate was 37.5%, and the drug-loading rate was 1.29%. After drug loading, the hybrid gold nanorod@MOF@doxorubicin (GMD) solution was irradiated with an 808 nm laser for 5 min prior to placement. The amount of the drug released was enhanced by laser irradiation (Figure 2B). The results showed that near-infrared irradiation promoted drug release, converted light energy into heat, and further promoted drug release.

### 2.3. Photothermal Conversion Effect

**Figure 3:**
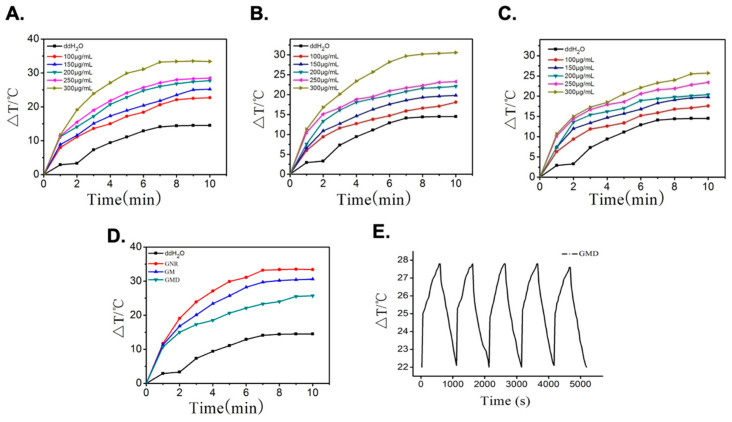
(**A**) Temperature profiles of GNR solution with different concentrations under laser irradiation; (**B**) temperature profiles of GM solution at different concentrations under laser irradiation; (**C**) temperature profiles of GMD solution with different concentrations under laser irradiation; (**D**) temperature profiles of GNR, GM, and GMD at a concentration of 300 μg/mL; (**E**) temperature cycling process of GMD solution in every 10-min period of radiation and 10-min period of cooling exposed to 808 nm laser.

The photothermal conversion characteristics of gold nanorods are important for tumor treatment [20]. As the GNR nanomaterial concentration increased, the temperature gradually increased (Figure 3A). When the GNR nanomaterial concentration was 300 μg/mL under an 808 nm laser with continuous irradiation for 10 min, the temperature rose by 32 °C, and then the temperature increase slowed down and gradually leveled off. Compared with GNR, GM, and GMD, temperature also increased with increasing concentration (Figure 3B,C); in GM, it rose by 30 °C, and in GMD, it rose by 26 °C. There was little difference between GNR, GM, and GMD in terms of photothermal conversion ability (Figure 3D). This could be caused by the gradual decrease in the GNR content of the three types of nanomaterials. 

To further explore GMD photostability, it was irradiated using an 808 nm laser for 10 min before being cooled to room temperature. After five cycles of near-infrared illumination, the temperature of each cycle remained almost unchanged, proving that GMD had good photostability (Figure 3E). This increased the efficiency of thermal therapy in tumor tissues.

### 2.4. Cytotoxicity Analysis

**Figure 4:**
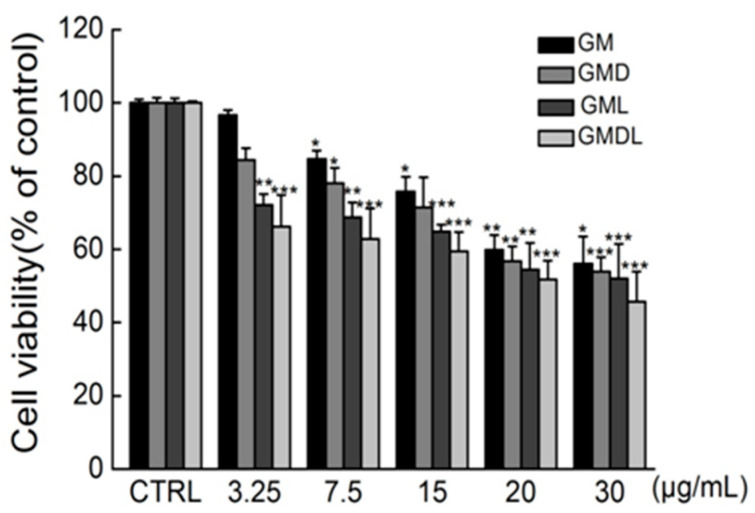
The effect of single administrations of gold nanorod@MOF (GM), gold nanorod@MOF@doxorubicin (GMD), gold nanorod@MOF@laser (GML), and gold nanorod@MOF@doxorubicin@laser (GMDL) at different concentrations for 24 h on the survival rate of A549 cells (* *p* < 0.05, ** *p* < 0.01, *** *p* < 0.001 vs. control group).

To verify the toxicity of the nanomaterials against cancer cells, the dose concentration of nanomaterials in A549 cells was screened using a 3-(4,5-dimethylthiazol-2-yl)-2,5-diphenyltetrazolium bromide (MTT) assay. GM was administered at a concentration range of 3.25–30 μg/mL, and the cell survival rate decreased gradually with increasing nanomaterial concentration, indicating that the nanomaterials had a concentration-dependent effect on A549 cells (Figure 4). When the nanomaterial concentration was 30 μg/mL, the cell survival rate was as low as 56.13 ± 7.40% (*p* < 0.05), indicating that GM nanomaterials at this concentration can significantly inhibit A549 cell proliferation. 

GMD was administered in a concentration range of 3.25–30 μg/mL, and cell survival rates decreased gradually with increasing GMD nanomaterial quantities, indicating that it could effectively induce A549 cell death. When the drug concentration was 30 μg/mL, the cell survival rate decreased to 53.98 ± 3.84% (*p* < 0.001).

GM nanomaterials have photothermal effects; therefore, the MTT method was used to explore their effects on A549 cell viability in combination with near-infrared light. After irradiation with an 808 nm laser with a power of 1 W·cm^−2^, the cell survival rate gradually decreased with increasing nanomaterial concentrations. When the drug concentration was 30 μg/mL, the cell survival rate decreased to 52.07 ± 9.44% (*p* < 0.001). At a specific nano-carrier concentration, the survival rate of cells treated with combined near-infrared light decreased by 1.91% compared with GM nanocarriers treated with cells alone. Thus, GM nanomaterials had a more significant effect on apoptosis induction when combined with near-infrared light.

The MTT assay was used to explore the effect of near-infrared light combined with GMD nanomaterials on A549 cell viability. After irradiation by an 808 nm laser with a power of 1 W·cm^−2^, the cell survival rate gradually decreased with increasing nanomaterial concentration. When the drug concentration was 30 μg/mL, the cell survival rate decreased to 45.67 ± 8.36% (*p* < 0.001), indicating that the apoptosis induced by GMD nanocarrier materials was significantly enhanced after combined treatment with near-infrared light. The cell survival rate was 51.81 ± 5.09% when the concentration was 20 μg/mL (*p* < 0.001), so the GMDL concentration was selected as 20 μg/mL in the follow-up experiment. After the cells were treated with different concentrations of GM, gold nanorod@MOF@laser (GML), GMD, and gold nanorod@MOF@doxorubicin@laser (GMDL) nanocarriers, cell survival gradually decreased with increasing nanocarrier concentrations (Figure 4). Under specific concentration conditions, the relationship between the cell survival rates of the four nanocarriers was as follows: GM < GMD < GML < GMDL. This was consistent with the results of the photothermal effect of nanocarrier materials and further proved the previously verified experimental results.

### 2.5. Hoechst 33342 Staining Was Used to Detect Cell Apoptosis

**Figure 5:**
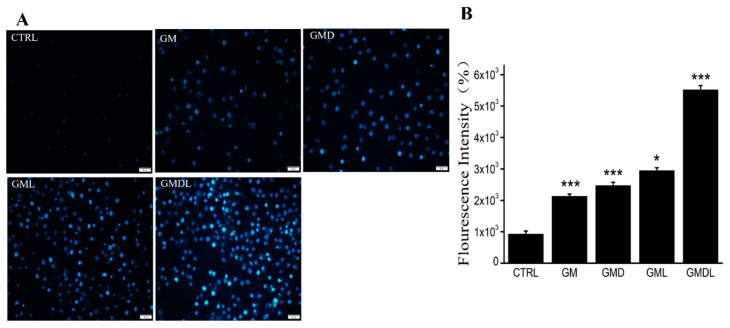
(**A**) Hoechst 33342 staining method, scale bar = 50 μm; (**B**) analysis using ImageJ (* *p* < 0.05, *** *p* < 0.001).

According to the above results, the synthesized nanomaterials induced cell injury and apoptosis (Figure S2). Therefore, Hoechst 33342 staining was used to further verify cell apoptosis (Figure 5A). The control group appeared uniform, faint, and dark blue in color. A small portion of the GM group cells underwent apoptosis, and their brightness was slightly dimmer than that of the control group. GMD group cells were similar. In the GML group, cell brightness increased significantly, indicating that more cells underwent apoptosis. The GMDL group showed the highest brightness and the most significant apoptosis among the five groups. ImageJ software (version: 1.51; National Institutes of Health, Bethesda, MD, USA) was used to analyze cell fluorescence intensity in each group (Figure 5B), and the results were consistent with the photographs and flow cytometry detection results. Therefore, we showed that GMDL had the most significant effect on the induction of apoptosis in A549 cells.

### 2.6. Mitochondrial Transmembrane Potential in A549 Cells

**Figure 6:**
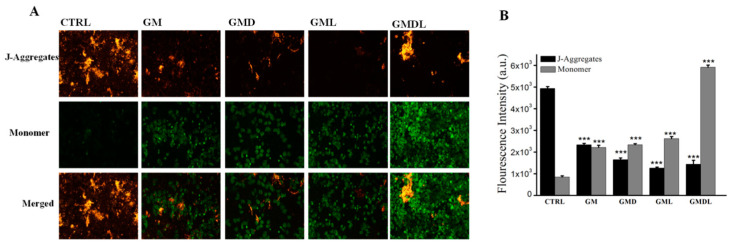
(**A**) Enhancement of mitochondrial membrane potential in A549 cells induced by GM, GMD, GML, and GMDL nanocarrier materials; scale bar = 50 μm. (**B**) analysis using ImageJ (*** *p* < 0.001).

Because GMDL can effectively induce apoptosis, we explored its mechanism of action. The mitochondrial transmembrane potential of A549 cells was determined (Figure 6A). The CTRL group consisted of cells in the normal state, and the mitochondria were undamaged; therefore, the transmembrane potential was normal. At this time, the fluorescent probe JC-1 entered the mitochondria and formed polymeric substances, thus showing enhanced red fluorescence, while green fluorescence was weak. When treated with GM, GMD, GML, and GMDL for 24 h, A549 cells were damaged, and apoptosis was induced. At this time, the mitochondrial transmembrane potential was depolarized, resulting in green fluorescence. Compared with the CTRL group, the GM, GMD, GML, and GMDL groups eventually showed reduced intracellular mitochondrial transmembrane potential, exhibiting weaker red and stronger green fluorescence (Figure 6A). Compared with the GM, GML, and GMD groups, green fluorescence was lower to some extent, indicating that the degree of cell damage was lower, whereas the green fluorescence intensity of the GMDL group was enhanced, indicating a greater degree of cell damage. ImageJ was used for the quantitative analysis of fluorescence intensity (Figure 6B), and the results were consistent with those of fluorescence microscopy. Overall, the GMDL group exhibited the strongest induction of apoptosis in A549 cells.

### 2.7. Intracellular Ca^2+^ Influx Concentration

**Figure 7:**
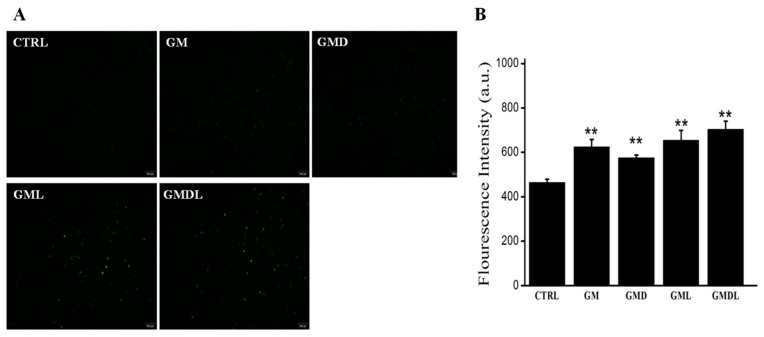
(**A**) GM, GMD, GML, and GMDL nanomaterials significantly enhanced the influx of Ca^2+^ in A549 cells; scale bar = 100 μm; (**B**) analysis using ImageJ (** *p* < 0.01).

In the control group, A549 cells were normal with intact cell membranes; therefore, the Fluo-4AM fluorescence probe could not enter the cells, showing weak green fluorescence (Figure 7A). The other drug administration groups induced A549 cell apoptosis to varying degrees; thus, cell membrane permeability was enhanced, and the Fluo-4AM probe could enter the cells and bind to excessive intracellular Ca^2+^. Compared with the control group, the Fluo-4AM probe showed different degrees of green fluorescence. Compared with the GM group, the GML and GMD groups showed varying degrees of green fluorescence, while the green fluorescence in the GMDL group was significantly enhanced and stronger than that in the control group, indicating that the range of apoptotic damage induced by the nanomaterial was the most significant. Simultaneously, the intracellular Ca^2+^ concentration was also the highest, showing strong green fluorescence. ImageJ was used for quantitative analysis of green fluorescence (Figure 7B), and the results were consistent with those obtained by fluorescence microscopy. Therefore, GMDL induced A549 cell apoptosis most substantially, with an increase in abnormal Ca^2+^ flow.

### 2.8. Intracellular Reactive Oxygen Species (ROS) Content in A549 Cells

**Figure 8:**
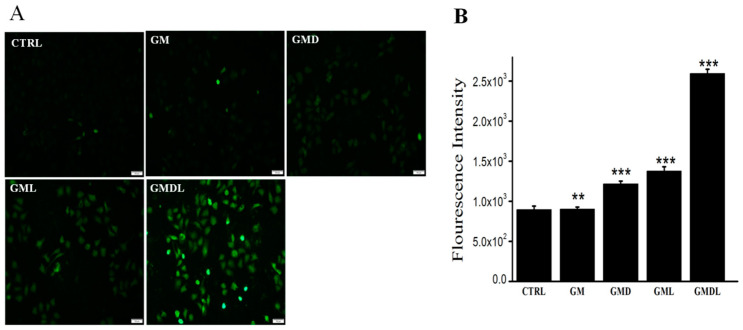
(**A**) Reactive oxygen species (ROS) overexpression in A549 cells induced by GM, GMD, GML, and GMDL nanocarrier materials; scale bar = 100 μm; (**B**) analysis using ImageJ (** *p* < 0.01, *** *p* < 0.001).

The control group consisted of A549 cells with normal physiology and intracellular ROS content; therefore, no obvious green fluorescence was observed (Figure 8A). The other drug administration groups showed obvious green fluorescence due to the drugs damaging the A549 cells, resulting in excessive ROS production, which further oxidized the hydrolyzed DCFH-DA fluorescent probe into fluorescent DCF, thus presenting strong green fluorescence. Compared with the GM and GMD groups, green fluorescence was weak, indicating a low degree of damage to cells, whereas green fluorescence was significantly enhanced in the GML and GMDL groups, especially the GMDL group, which was the highest. ImageJ was used for quantitative analysis of green fluorescence (Figure 8B), and the results were consistent with those obtained by fluorescence microscopy. Therefore, we found that the GMDL group effectively disrupted the balance between the oxidative and antioxidant systems in cells, resulting in the production of large amounts of ROS.

### 2.9. Caspase-3/-8/-9 Enzyme Activity

**Figure 9:**
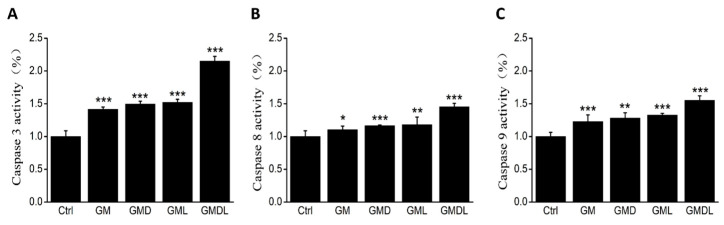
(**A**) Changes in caspase-3 enzyme activity for GM, GMD, GML, and GMDL nanomaterials after 24 h incubation in A549 cells (*** *p* < 0.01); (**B**) changes in caspase-8 enzyme activity for GM, GMD, GML, and GMDL nanomaterials after 24 h incubation in A549 cells (* *p* < 0.05, ** *p* < 0.01, *** *p* < 0.001); (**C**) changes in caspase-9 enzyme activity for GM, GMD, GML, and GMDL nanomaterials after 24 h incubation in A549 cells (** *p* < 0.01, *** *p* < 0.001).

The effects of GM, GMD, GML, and GMDL on caspase-3, caspase-8, and caspase-9 activities in A549 cells were determined using a specific fluorescence assay. The caspase-3 content was upregulated after treatment with the nanomaterials (*p* < 0.01; Figure 9A). Caspase-3 is an executor of apoptosis; in comparison, the untreated cell group showed upregulated caspase-8 (*p* < 0.01; Figure 9B) and caspase-9 (*p* < 0.01; Figure 9C) levels. These results indicated that the cells were activated by endogenous and exogenous apoptotic pathways, which promoted the programmed apoptotic system.

### 2.10. Western Blotting of A549 Cell Proteins

**Figure 10:**
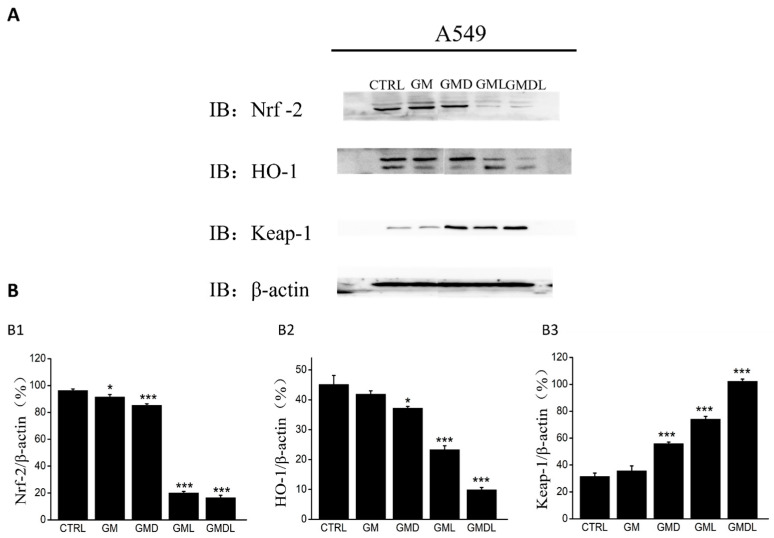
(**A**) Effects of GM, GMD, GML, and GMDL nanomaterials on the expression of oxidative stress signaling pathway-related proteins in A549 cells; (**B**) analysis using ImageJ in Nrf2 (**B1**), HO1 (**B2**), Keap1 (**B3**) protein expression effects (* *p* < 0.05, *** *p* < 0.001).

Keap1/Nrf2 is a crucial antioxidant signaling pathway that controls cell apoptosis. Here, we primarily studied protein content changes in Keap1, Nrf2, HO1, and their downstream signaling factors after administration. Compared with the control group, Nrf2 and HO1 protein expression was downregulated and Keap1 expression was upregulated after GM treatment (Figure 10). After drug and photothermal GML and GMDL administration, Nrf2 (*p* < 0.001), HO1 (*p* < 0.001), and Keap1 (*p* < 0.001) expression levels decreased significantly, and the effects were significantly stronger than those in the GM and GMD groups without photothermal treatment. These results indicated that anti-tumor nanomaterials, along with their synergistic photothermal effects, can activate antioxidase-related protein expression during cancer therapy.

## 3. Discussion

Nanodrug carriers based on gold nanorods are a new and significant research topic [18,19,20]. GNR nanomaterials were characterized using their solution color, UV–visible absorption spectra, and TEM, confirming their successful synthesis [21,22,23]. They were then characterized using TEM, UV–visible absorption spectroscopy, Fourier transform infrared spectroscopy, and STEM-EDS specta [24,25,26,27], which proved the successful synthesis by coating Cu-MOF on the GNR surface. Finally, GMD was characterized by transmission electron microscopy and STEM-EDS, and the results proved that GMD nanomaterials have drug-carrying capacity, as we successfully loaded DOX (Figure S3). 

We found that GMD nanomaterials had horseradish peroxidase (HRP) activity at a pH of 5 and a temperature of 37 °C (Figure S4). In normal cells, oxidation and antioxidant systems maintain a fairly balanced state [28,29,30], while an increase in pro-oxidation levels or a decrease in antioxidant capacity can lead to an increase in ROS levels, resulting in a cascade of changes [31,32]. ROS levels are higher in tumor cells than in normal cells, resulting in oxidative stress [33,34]. Therefore, GMD nanomaterials exert their biological functions by elevating ROS levels (Figure 9). Subsequently, we examined the photothermal effects of the GM and GMD nanomaterials, realizing favorable recyclability of the photothermal conversion in tumor therapy. The drug loading time was screened, and we found that maximum drug loading could be achieved with ultrasound for 30 min. The GMD drug loading and encapsulation rates were 1.29% and 37.5%, respectively. We also explored the drug release ability and conditions and determined that the amount of the drug released could be increased under near-infrared light, which is conducive to sustained drug release. Ultimately, the GMD nanomaterials we constructed not only combined a photothermal effect with that of a chemotherapeutic drug but also had an oxidation function.

To further verify its anti-tumor activity through in vitro cell experiments, GM, GMD, GML, and GMDL nanomaterials were shown to reduce A549 cell survival by the MTT method, especially after 30 μg/mL GMDL treatment. The apoptosis rate was the highest, and the cell survival rate was only 45.67 ± 8.36%. The free DOX was used to test the cytotoxicity (Figure S5). The calculated IC_50_ of DOX is 1.15 μg/mL. We also calculated the IC_50_ of DOX in the DOX-loaded system (GMD, GMDL groups); the drug IC_50_ was 0.41 μg/mL for GMD and 0.29 μg/mL for GMDL. This combinatorial therapy effectively reduced the IC_50_ of the drug. It may considerably increase the sensitivity of cells to DOX, with about a 4.0-fold reduction. Apoptosis was investigated by flow cytometry (Figure S2). The above-mentioned nanomaterials induced apoptosis to varying degrees, especially in the GMDL group, where it was as high as 90%, consistent with the MTT results. The cell membranes are broken after cell injury and apoptosis induced by the nanomaterial, allowing the Hoechst 33342 fluorescent probe to easily pass through the biofilm and bind to the double-stranded DNA embedded in the nucleus, forming obvious bright blue fluorescence. Through experimental observations, we found that these nanomaterials produced bright blue fluorescence, which was particularly significant in the GMDL group. For in vivo application, the size distribution of the GMD system was evaluated by DLS (Figure S6). A range of 140–950 nm is not commonly associated with enhanced permeability and retention (EPR effect) in tumors. However, the large size (up to 2600 nm) of the drug system also exhibited specific biodistribution in mice [35]. The particle suspension can be injected via the tail vein, with larger particles showing accumulation in the lung tissue. Therefore, the A549 cell line in our system was used to assess the feasibility in tumor treatment. This could help us to establish animal experiments for lung cancer treatment in the future.

We also found that intracellular Ca^2+^ flow increased after treatment with nanomaterials, and the intracellular mitochondrial transmembrane potential and reactive oxygen species levels improved, showing significant performance in the GMDL group in various experiments. Additionally, nanomaterials reduced Nrf2 and HO1 protein expression levels and regulated Caspase-3/-8/-9 and Keap1 levels. 

**Scheme 1:**
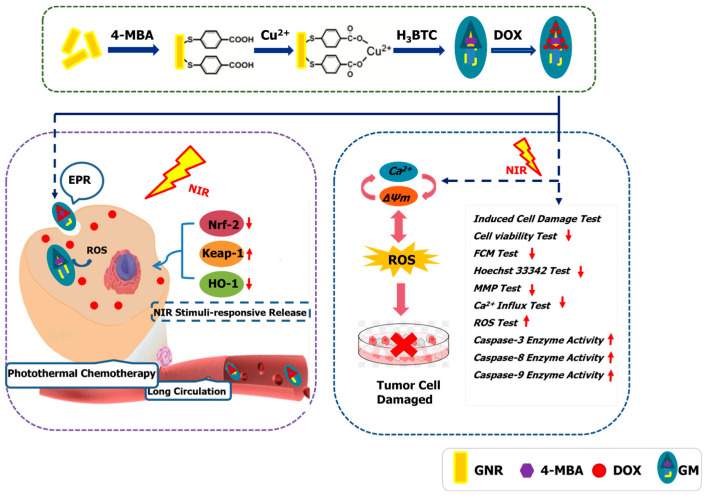
Schematic experiments of GMDL nanomaterials in tumor treatment. Under near-infrared light irradiation, GMD nanomaterials may induce cell apoptosis; they reduced cell viability by lowering intracellular Ca^2+^ influx, destroying mitochondrial membrane potential stability, elevating reactive oxygen species level, and upregulating the expression of caspase-3/8/9 enzyme activity.

Overall, this GMDL nanomaterial can induce A549 cell apoptosis, indicating that it can improve combinatorial cancer therapy and enhance cancer treatment (Scheme 1).

## 4. Materials and Methods

### 4.1. Chemicals and Characterization

All analytical-grade chemicals used in this study were purchased from Aladdin Chemistry Co., Ltd. (Shanghai, China) and used directly without purification. HRP (≥300 U·mg^−1^) was purchased from Shanghai Yuanye Bio-Technology Co., Ltd. (Shanghai, China). H_2_O was purified using a Milli-Q water purification system (Millipore, St. Louis, MO, USA). The UV–Vis and fluorescence spectra of the nanomaterials were collected using a UV-2700 spectrophotometer (Shimadzu Corp., Kyoto, Japan) and an RF-6000 fluorescence spectrophotometer (Shimadzu Corp.), respectively. FTIR spectra were acquired using a VERTEX 80VFT-IR spectrometer (Bruker Daltonik GmbH, Bremen, Germany). The TEM images were obtained using a JEM-2100 microscope (JEOL Ltd., Tokyo, Japan) at 200 kV. The particle sizes were determined using the Dynamic Light Scattering (DLS) method with a Zetasizer Nano ZS 90 (Malvern Panalytical, Malvern, UK).

### 4.2. Preparation and Characterization of Nanomaterials

#### 4.2.1. Synthesis of GNRs

GNRs were prepared using a previously published method [18]. Firstly, 1.8 mL of 0.2 M cetyltrimethyl ammonium bromide (CTAB) solution and 72 μL of 10.0 mM tetrachloroauric acid (HAuCl_4_·4H_2_O) were slowly poured into the centrifuge tube and gently stirred evenly. Then, 4.1 μL AgNO_3_ solution (4.0 mM) was dropped into the centrifuge tube evenly while shaking. Next, 5.0 μL (1.0 M) hydrochloric acid solution was added to the centrifuge tube, and the pH of the solution was adjusted to about 1. We then added 94.5 μL (0.1 M) hydroquinone solution to the centrifuge tube and stirred it vigorously for 15 min. Stirring was stopped when the solution became colorless. After that, 0.7 μL of NaBH_4_ (10.0 mM) was added immediately while the tube was in an ice bath, and the mixed solution was placed in a 27 °C water bath overnight. The solution was then centrifuged at 9000 rpm for 15 min and cleaned twice to obtain a GNR stock solution (1 mg/mL).

#### 4.2.2. Synthesis of MOF-Capped Gold Nanorod

A quantity of 100 μL of 4-mercaptobenzoic acid (4-MBA) ethanol solution (5 mg/mL) was added to 2 mL GNR stock solution with agitation by vigorous shaking for an additional 2 h in a 27 °C water bath. After the reaction was completed, 10 μL copper acetate aqueous solution (10 mg/mL) was added to the above solution, and it was sonicated for 10 min. The solution was mixed well with the following added in order: 1 mL H_2_O, 1 mL trimesic acid–N,N-dimethylformamide solution (*v*:*v* = 1:1). This was subjected to ultrasonication for 10 min. This was then incubated for 30 min in a 55 °C water bath, and GM was obtained via centrifugation at 9000 rpm for 10 min before being stored in ethanol at 4 °C. 

#### 4.2.3. DOX Loading

MOF-capped gold nanorods hybrids were obtained by mixing the prepared doxorubicin (DOX) aqueous solution (100 μg/mL) with the GM nanomaterials suspension (1 mg/mL) via sonication for 30 min. According to the carrying amount and drug-loading formula, the encapsulation rate and the drug-loading rate were calculated via UV–Vis absorption spectroscopy. After centrifugation and discarding the supernatant, the gold nanorod@MOF@DOX (GMD) suspension was dissolved in water for later use.

### 4.3. Photothermal Performance of Nanomaterials

To measure the photothermal conversion performance of different nanomaterials, GNR, GM, or GMD with different concentrations (100, 150, 200, 250, and 300 μg/mL) in 1 mL aqueous solution were irradiated with near-infrared light (808 nm laser, 1.0 W·cm^−2^) for different amounts of time, and a temperature sensor was used to record the temperature change per minute. 

### 4.4. Drug Loading and Release 

The DOX-loading ability was evaluated according to the incubation time of the drug in the GM suspension. The GM deposit (100 μg) was resuspended in 1 mL of DOX solution (70 μg/mL) by ultrasonication for different time periods (10–40 min). After incubation, the GM–DOX suspension was centrifuged at 9000 rpm for 10 min. The absorbance intensity of the supernatant was measured using a UV–Vis spectrophotometer. A standard curve was recorded in a concentration range of 10–60 μg/mL DOX aqueous solution using the UV–Vis spectrophotometer. The loading capacities and encapsulation efficiency of the GMD hybrids were measured using the standard curve method. 

Release behavior was monitored in a simulated tumor environment. GMD hybrids were dissolved in citric acid buffer solution (pH 5) with a temperature of 37 °C for 30 min. After centrifugation at 9000 rpm for 10 min, the supernatant was analyzed using a UV–Vis spectrophotometer. For photodynamic release, the GMD sample was exposed to an 808 nm laser for 5 min and incubated for 25 min for detection. 

### 4.5. Peroxidase-like Activity

In the positive control group, 930 μL of citric acid buffer (50 mM, pH 5), 10 μL of HRP (1.6 ng/mL), 20 μL of 3,3′,5,5′-tetramethylbenzidine (TMB), and 40 μL of H_2_O_2_ (4 mM) were added successively. Next, in the experimental group, 100 μL of GMD (0.2 mg/mL), 20 μL of TMB, and 40 μL of H_2_O_2_ (4 mM) were added in order to 840 μL citric acid buffer (50 mM, pH 5). Then, in the negative control group, 20 μL of TMB and 40 μL of H_2_O_2_ (4 mM) were added successively to 940 μL of citric acid buffer (50 mM, pH 5). The reaction temperature was 37 °C, and the reaction time was 15 min. UV–Vis absorption measurements were also performed. A typical reaction involves the catalytic reduction of H_2_O_2_ to water, coupled with TMB oxidation (Equation (1)). The optical density at 652 nm was used to calculate relative activity.

(1) $$H_{2}O_{2}+TMB+H^{+}\overset{HRP/GMD}{\to }2H_{2}O+{TMB}_{ox}$$

### 4.6. Cell Culture

A549 cells (low differentiation; passages < 10; CRL-1721; ATCC, Manassas, VA, USA) derived from cancerous human lung alveolar basal epithelial cells were used. They were cultured in Dulbecco’s modified eagle medium (DMEM; Thermo Fisher Scientific, Waltham, MA, USA) and supplemented with 10% (*v*/*v*) horse serum (HS; Kangyuan Biology, Tianjin, China), 5% (*v*/*v*) fetal bovine serum (FBS; Kangyuan Biology, Tianjin, China), 100 μg/mL penicillin, or 100 μg/mL streptomycin (Invitrogen, Carlsbad, CA, USA). The cells were incubated under a humidified atmosphere containing 5% CO_2_ and 95% air at 37 °C. The media were replaced every 2 d. The cells were considered ready for treatment when they reached 75% confluence.

### 4.7. MTT Assay

A549 cells were inoculated in 96-well plates at a density of 2 × 10^4^ cells per well at 37 °C under 5% CO_2_. Different concentrations of the nanomaterials were added and co-incubated. After 24 h, cells were digested with 0.25% (*w*/*v*) trypsin and centrifuged. Next, 5 mg/mL MTT solution was added to the cells, and they were incubated for 4 h at 37 °C in the dark. Then, 100 μL of DMSO was added to dissolve the formazan crystals. The MTT assay was then repeated as described above. A microplate reader (Infinite F200 Pro; Tecan Group AG, Männedorf, Switzerland) was used to measure optical density (OD) at 540 nm. In the laser exposure experiments, GML and GMDL samples were added with different concentrations of GM and GMD for 24 h, irradiated with a near-infrared laser (808 nm) of 1 W/cm^2^ for 5 min, and cultured for 3 h. The MTT assay was then conducted. 

### 4.8. Analysis of Apoptosis Morphology

The A549 cells were treated with GM, GMD, GML, or GMDL for 24 h. The laser exposure time was 5 min after incubation. The cells were washed with phosphate buffer and centrifuged to remove the supernatant. The Annexin V-FITC/PI kit dye was used for 30 mins at 4 °C in the dark. The stained cells were collected by centrifugation, and the excess staining fluid was removed. The cells were suspended in phosphate buffer, and the fluorescence of each cell was analyzed using flow cytometry (CytoFLEX, Beckman Coulter, Brea, CA, USA). 

### 4.9. Nuclear Apoptosis Measurement

A549 cells were treated with the different nanomaterials for 24 h. At 37 °C in the dark, 5 μg/mL Hoechst 33342 (Beyotime, Shanghai, China) was added to stain the cells and hatched for 20 min. The cells were then washed three times with phosphate buffer. Morphological changes in apoptotic cells were photographed using a fluorescence microscope (×10; CCD camera; IX73; Olympus, Tokyo, Japan). ImageJ software was used for the quantitative analysis of the fluorescent images.

### 4.10. Analysis of Mitochondrial Membrane Potential (MMP)

A549 cells were treated with the different nanomaterials for 24 h. At 37 °C in the dark, 10 μg/mL JC-1 (Beyotime, Shanghai, China) staining solution was added to stain the cells and hatched for 20 min. The cells were then washed three times with phosphate buffer. Red and green fluorescence were measured using a fluorescence microscope. 

### 4.11. Intracellular Ca^2+^ Concentration

A549 cells were treated with the different nanomaterials for 24 h. At 37 °C in the dark, 5 μM Fluo-4 AM (Fluo-4 acetoxymethyl ester) staining solution was added to stain the cells and hatched for 20 min. The cells were then washed thrice with phosphate buffer and analyzed using a fluorescence microscope. 

### 4.12. Intracellular ROS

Intracellular ROS levels were detected using 2′,7′-dichlorofluorescein diacetate (DCFH-DA) (Beyotime Biotechnology, Shanghai, China). Briefly, the A549 cells were treated with different nanomaterials for 24 h. At 37 °C in the dark, 10 μM DCFH-DA solution was added to stain the cells and hatched for 30 min. The cells were then washed three times with phosphate buffer. Morphological changes in the cells were photographed using a fluorescence microscope. Intracellular green fluorescence intensity was detected using a microplate reader.

### 4.13. Caspase 3/8/9 Activity Assay

A549 cells were treated with various experimental drugs for 24 h. Digested cells were washed with phosphate buffer and centrifuged to remove the supernatant. The protein content of the cell lysates was determined according to the BCA protein assay kit. Extracted protein was also added to the buffer and caspase 3/8/9 assay substrate and incubated for 4 h at 37 °C in the dark. Absorbance at 400 nm was measured using a microplate reader.

### 4.14. Western Blotting

A549 cells treated with different nanomaterials were lysed and centrifuged for 10 min at 12,000 rpm (4 °C). The total protein content of the supernatant was determined using a bicinchoninic acid (BCA) protein assay kit (Biosynthesis Biotechnology, Beijing, China). The proteins were loaded onto concentrated gel and 10% sodium dodecyl sulfate-polyacrylamide gel, separated by electrophoresis (SDS-PAGE), and transferred to a 0.45 μm polyvinylidene fluoride (PVDF) membrane (Invitrogen, Carlsbad, CA, USA). After methanol activation for 1 min, 5% (*v*/*v*) bovine serum albumin (BSA) was blocked with Tris-buffered saline and Tween-20 (TBST) at 25 °C for 2 h. Then, the membranes were probed with primary antibodies, anti-β-actin (bs-0061R), Nrf2 (bs-1074R), keap1 (bs-3648R) (all from Biosynthesis and Technology, Beijing, China), and HO1 (ab13248; Abcam, Cambridge, UK), respectively, overnight at 4 °C. TBST was used to wash them three times for 10 min each. The blots were then incubated with the corresponding HRP-conjugated secondary antibodies (HRP-labeled goat anti-rabbit IgG antibody 1:1000, CST, USA) for 1 h. Relative protein band intensities were detected using enhanced chemiluminescence (ECL, C05-07004, Biosynthesis Biotechnology Co., Ltd., Beijing, China) and analyzed using a fully automated chemiluminescence imaging analysis system (Tanon 5200, Teneng Technology Co., Ltd., Shanghai, China).

### 4.15. Statistical Analysis

All data are presented as the mean ± SEM. The statistical significance of the differences between two groups was analyzed by Student’s *t*-test or one-way analysis of variance (ANOVA). Statistical significance was set at *p* < 0.05.

## 5. Conclusions

Nanodrug carriers based on gold nanorods are a new and exciting research topic. In this study, we successfully constructed a multifunctional gold nanorod composite drug carrier. Based on gold nanorods, a Cu-MOF layer was wrapped around the drug carrier. Such a design not only prevented the photothermal effect of the nanorods from being lost but also maintained the enzyme-like activity of the Cu-MOF structure. Compared with gold nanorods, the drug carrier GMD ability decreased, but the difference was small. After the gold nanorods were wrapped with the Cu-MOF layer acting as the basic skeleton, the near-infrared photothermal conversion efficiency was unaffected, allowing them to be used in tumor therapy. GM nanocrystalline materials exhibit HRP activity and can exert biological functions by adjusting the ROS levels in tumor cells. GMD nanocarrier material activity, combined with near-infrared light (GMDL) irradiation of tumor cells and its related mechanism, was found to have anti-tumor activity at the cellular level. GMDL reduces Nrf2 and HO1 protein expression. Caspase-3/-8/-9 and Keap1 levels were regulated, suggesting that the nano-carrier may induce tumor cell apoptosis damage by mediating Nrf2 or Keap1. Such nanocarrier materials could not only play an anti-tumor role but could also be combined with physical and chemical therapies to realize improved elimination of tumor cells, resulting in functional enhancement. Therefore, GMDL nanocarrier materials have great potential for development in anti-tumor research in the future.

## Data Availability

All data can be found in the manuscript.

## Supplementary Materials

The following supporting information can be downloaded at: https://www.mdpi.com/article/10.3390/molecules29102384/s1, Figure S1: Standard cure of DOX aqueous solution; Figure S2: Effects of GM, GMD, GML, and GMDL nanomaterials on the induction of apoptosis in A549 cells; Figure S3: EDS spectrum and TEM image of GMD nanomaterials; Figure S4: The enzyme-like analysis of GMD; Figure S5: The effect of single administrations of DOX on the survival rate of A549 cells; Figure S6: The size distribution of GMD by DLS.
