# Supplementary material for: Metal–Organic Framework-Capped Gold Nanorod Hybrids for Combinatorial Cancer Therapy
Source: Molecules. 2024 May 18;29(10):2384. doi: 10.3390/molecules29102384 (PMC11124105; doi:10.3390/molecules29102384)
Supplement: Supplementary file 1 [file molecules-29-02384-s001.zip › molecules-3013770-supplementary.pdf]

Supporting Information

Metal-Organic Framework-Capped Gold Nanorod Hybrids for Combinatorial Cancer Therapy

Chong Zhao [1,†], Hongxiang Liu [1,†], Sijun Huang [1], Yi Guo [1,*] and Li Xu [1,*]

[1] Key laboratory for Molecular Enzymology and Engineering, the Ministry of Education, National Engineering Laboratory for AIDS Vaccine, School of Life Sciences, Jilin University, Changchun 130012, China; zhaochong23@mails.jlu.edu.cn (C.Z.); hxliu15@mails.jlu.edu.cn (H.L.); huangsj17@mails.jlu.edu.cn (S.H.); guoyi@jlu.edu.cn (Y.G.); xuli@jlu.edu.cn (L.X.)

* Correspondence: E-mail: guoyi@jlu.edu.cn (Y.G.); xuli@jlu.edu.cn (L.X.), Tel: 86-431-85155226 (L.X.)

† These authors contributed equally to the work.

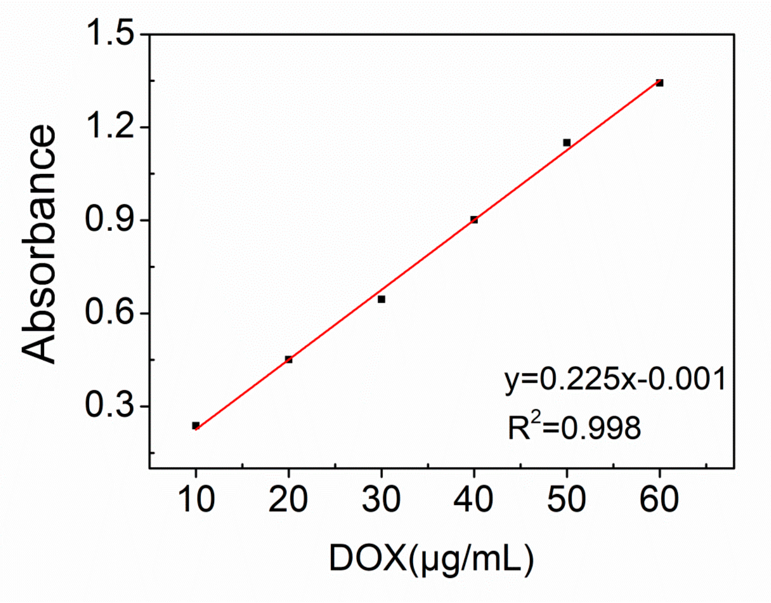

Figure S1. Standard cure of DOX aqueous solution.

GM and GMD alone can induce cell apoptosis by flow cytometry, and apoptosis rate can reach about 20% (Figure S2), however, when combined with near-infrared light, it can increase to more than 60%. These results prove that after exposure to light, photothermal plays a certain role in cell apoptosis.

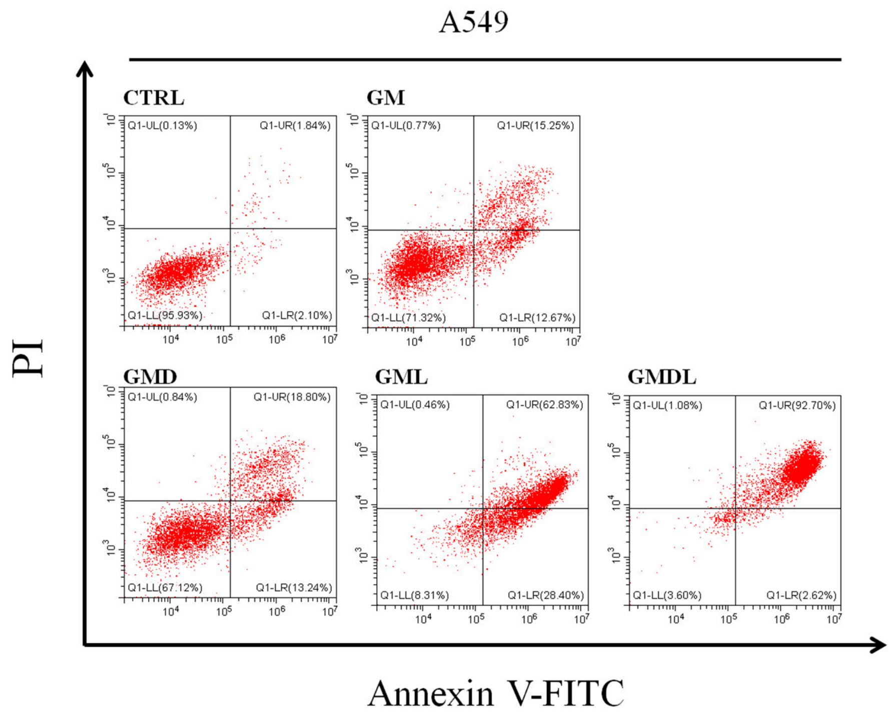

Figure S2. Effects of GM, GMD, GML and GMDL nanomaterials on the induction of apoptosis in A549 cells.

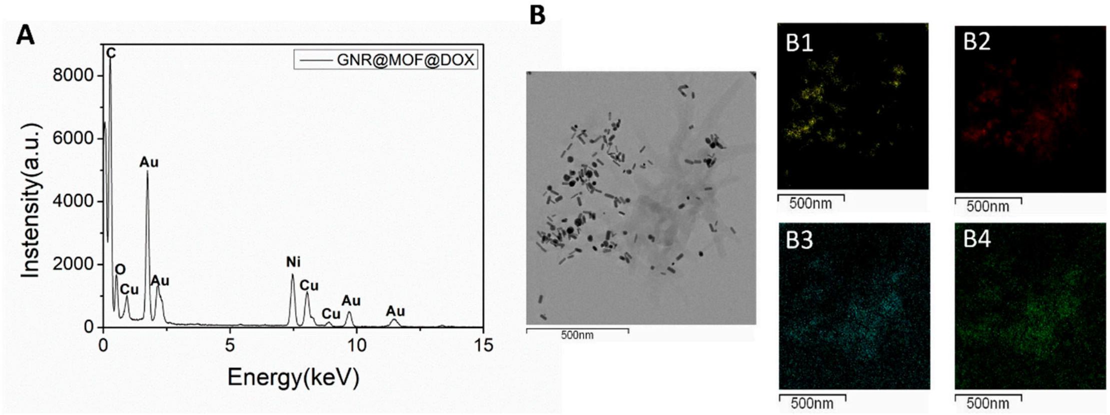

Figure S3. (A) EDS spectrum of GMD nanomaterials; (B) TEM image of GMD nanomaterials. STEM-EDS elemental mapping of GMD EDS analysis of GMD nanomaterials: Au (B1), Cu(B2), N(B3), O(B4).

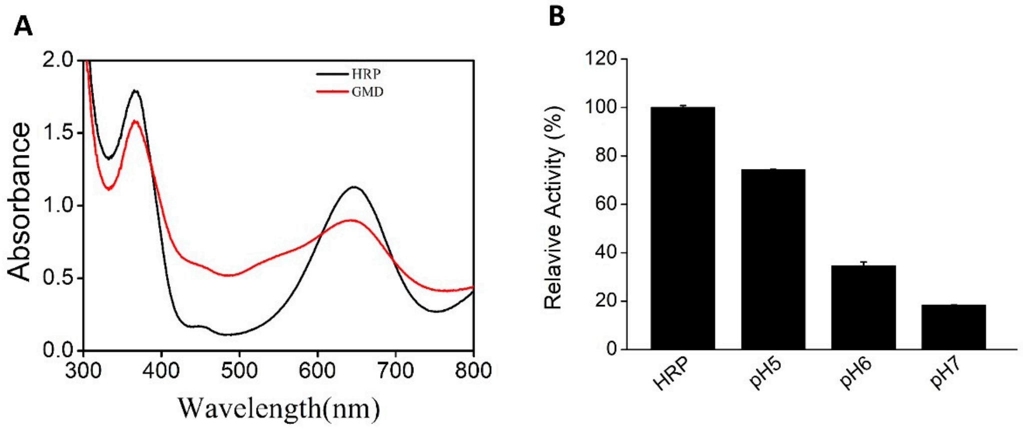

Figure S4. (A) UV-Vis absorption spectrum of GMD and HRP for the enzyme-like analysis in the presence of hydrogen peroxide and TMB in pH 5 and 37 °C; (B) Relative activity of pH effect on enzyme-like behavior.

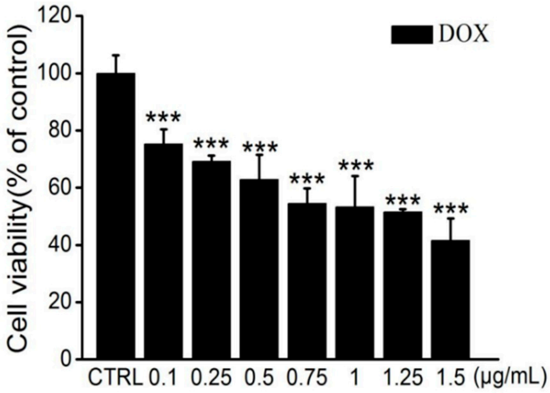

Figure S5. The effect of single administrations of DOX at different concentrations for 24 h on the survival rate of A549 cells. (***P <0.001 vs. control group)

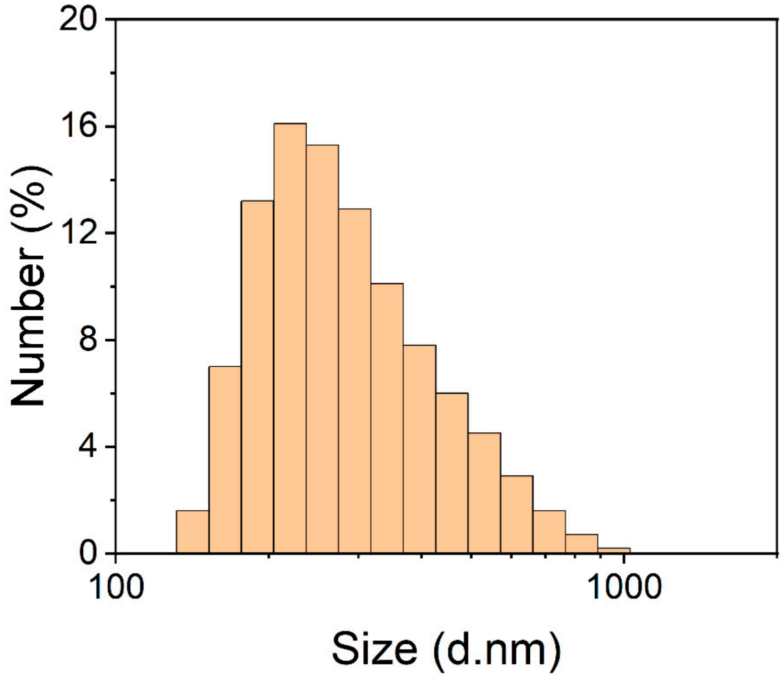

Figure S6. The size distribution of GMD by DLS.
